# C677T Polymorphism in the *MTHFR* Gene Is Associated With Risperidone-Induced Weight Gain in Schizophrenia

**DOI:** 10.3389/fpsyt.2020.00617

**Published:** 2020-07-02

**Authors:** Jingping Liao, Ning Wang, Mengying Ma, Tianlan Lu, Hao Yan, Weihua Yue

**Affiliations:** ^1^ Institute of Mental Health, The Sixth Hospital, Peking University, Beijing, China; ^2^ National Clinical Research Center for Mental Disorders & Key Laboratory of Mental Health, Ministry of Health (Peking University) & Chinese Academy of Medical Sciences Research Unit (No.2018RU006), Beijing, China; ^3^ School of Nursing, Peking University, Beijing, China; ^4^ Center for Biological Psychiatry, Beijing HuiLongGuan Hospital, Beijing, China; ^5^ PKU-IDG/McGovern Institute for Brain Research, Peking University, Beijing, China

**Keywords:** schizophrenia, risperidone medications, *MTHFR*, antipsychotic, weight gain

## Abstract

**Objective:**

To explore the association of methylenetetrahydrofolate reductase (*MTHFR*) C677T polymorphism with risperidone-induced weight gain.

**Methods:**

We analyzed the association between *MTHFR* C677T polymorphism and risperidone-induced weight gain in 356 schizophrenia patients. The patients were treated with risperidone for 8 weeks. The height and body weight of the patients were measured before and 8 weeks after risperidone treatment. Blood DNA was genotyped for *MTHFR* C677T polymorphisms.

**Results:**

We found a significant association between *MTHFR* C677T polymorphism and body mass index (BMI) change after 8-week risperidone treatment. The BMI of carriers with different genotypes of *MTHFR* gene increased over 2–8 weeks. After 8 weeks of risperidone treatment, BMI added value (kg/m^2^) of CC or CT genotype carriers was significantly higher than that of TT genotype carriers [CC (4.47 ± 1.09), CT (4.54 ± 1.27), TT (2.31 ± 0.75), F = 5.634, P = 0.001]. Based on whether the rate of weight gain from baseline at 8 weeks of treatment exceeded 7%, it was divided into a weight gain group (n = 61) and a non-weight gain group (n = 295). The C allele frequency was significantly different between the two groups (48.4% vs 32.4%, *χ^2^* = 11.342, P = 0.001).

**Conclusion:**

*MTHFR* C677T polymorphism was associated with risperidone-induced weight gain in Chinese Han population.

## Introduction

Schizophrenia is a chronic, complex, and severe mental disorder, affecting approximately 1% of the general population. Schizophrenia treatments are commonly accompanied by various adverse side effects (1). With the prevailing use of atypical antipsychotics or second-generation antipsychotics (SGAs), the adverse effects such as weight gain and metabolic abnormalities have become a major issue in the treatment of with SGAs. Antipsychotic-induced weight gain may not only adversely affect treatment adherence and clinical outcomes, but also is associated with reduced quality of life, social stigma, and greater cardiovascular morbidity and mortality (2–4).

Susceptibility to antipsychotic-induced weight gain varies substantially between individuals in ways that cannot be fully explained by differences in drug effects or other environmental factors. Thus, genetic factors are strongly implicated, and researches have been conducted to find possible associations between many genetic polymorphisms and antipsychotic-induced weight gain (5). The genetic factors which were commonly reported to be involved in antipsychotic-induced weight gain include polymorphisms in genes for *HTR2C* (6), *5-HT2A* (7, 8), *MC4R* (9), *Leptin* (10), *FTO* (11), and *BDNF* (12).

Genetic variants of the methylenetetrahydrofolate reductase (*MTHFR*) gene was also proposed as a potential candidate for antipsychotic-induced weight gain (13–15). *MTHFR* gene exerts an important role in folate/homocysteine metabolism that convert 5,10-methylenetetrahydrofolate to 5-methyltetrahydrofolate, which is used in methionine synthesis from homocysteine. The single-nucleotide polymorphisms (SNPs) rs1801133 (C677T) and rs1801131 (A1298C), two most important SNPs of *MTHFR* gene, are known to affect the function of enzymes and have shown potential clinical significance. The *MTHFR* gene C677T polymorphism can decrease their corresponding enzymes activities (16), which ultimately lead to enhanced plasma total homocysteine concentration and decreased folate concentration (17–19). Previous literatures have reported lower folate or higher homocysteine levels in obesity/overweight subjects compared with normal weight controls (20–22). It indicated that *MTHFR* C677T polymorphism might be one of genetic factors involved in antipsychotic-induced weight gain. However, the mechanisms underlying these observations remained unclear.

Recently, some studies investigated the association of *MTHFR* C677T polymorphism with antipsychotic-induced weight gain (15, 23). A previous research suggested that the *MTHFR* C677T polymorphism might, along with other genetic risk factors, provided a useful marker for the side effect of antipsychotic drug-induced weight gain (15). However, another study did not detect the influence of *MTHFR* C677T polymorphisms on antipsychotic-induced weight gain (23). Thus, these results are conflicting and the relationship between *MTHFR* C677T polymorphisms and antipsychotic-induced weight gain needs to be further clarified. Besides, the research about the association of *MTHFR* C677T polymorphism with weight gain caused by one single antipsychotic such as risperidone is rare. In the present study, we examined the association of the *MTHFR* C677T polymorphism with risperidone-induced weight gain in patients with schizophrenia.

## Materials and Methods

### Subjects

This study was an open clinical trial of risperidone monotherapy for 8 weeks. Oral administration was adopted, starting at 1 mg/d, and dosage was added within 2 weeks to reach the therapeutic dose of 2–6 mg/d. All participants were not allowed to use any other antipsychotics, antidepressants, anxiolytics, or mood stabilizers during the study period. If the patients have severe sleep disturbances, benzodiazepines can be used intermittently at night for short periods. Height and weight of the patients were measured before and 8 weeks after treatment, and body mass index (BMI; kg/m^2^) was calculated as weight (kg)/height (m)^2^. The Medical Ethics Committee of Institute of Mental Health, Peking University approved this study. This study was conducted in accordance with the Declaration of Helsinki. All participants were informed about the objectives and procedures and written informed consent was obtained.

All subjects were schizophrenics from outpatient and/or inpatient departments. Patients included in this research are required to meet the following inclusion criteria: (1) Chinese Han descents; (2) meeting the diagnostic criteria of schizophrenia on the basis of the Diagnostic and Statistical Manual of Mental Disorders, 4th edition (DSM-IV); (3) age ranged from18 to 65 years, gender unlimited; (4) total score of positive and negative symptom scale (PANSS) ≥ 60. The exclusion criteria were as follows: (1) pregnant, lactating or menopausal women; (2) patients with psychoactive substance abuse or other severe mental illness; (3) suffering from severe and unstable physical diseases such as diabetes, thyroid disease, hypertension.

### Genotyping

Five milliliters of peripheral blood samples from all subjects were collected in tubes containing EDTA as an anticoagulant and stored at 4°C. Genomic DNA was extracted from the samples using a Qiagen QIAamp^®^ DNA Mini Kit (Qiagen GmbH, Hilden, Germany) within one week, and stored at -70°C for later use. The *MTHFR* C677T polymorphism was genotyped by the direct DNA sequencing method. The sequences of the primers were as follows: 5′ AGC CCA GCC ACT CAC TGT TTT 3′ and 5′ CAG CGA ACT CAG CAC TCC A 3′. The PCR amplification was carried out in a 25 µl volume containing 10 mM Tris-HCl (pH 8.3), 50 mM KCl, 1.5 mM MgCl_2_, 200 µM of each dNTP, 0.4 µM of each primer, 1U of Taq DNA polymerase and 30–50 ng genomic DNA. The PCR amplification conditions were: an initial denaturation at 94°C for 5 min, followed by 35 amplification cycles at 94°C for 30 s, 64°C for 30 s, 72°C for 1 min, and then elongation at 72°C for 10 min. For SNP rs1801133, the PCR products were sequenced by DNA sequencing after cleaning the PCR product using BigDye Terminator Cycle Sequencing Ready Reaction Kit with Ampli-Taq DNA polymerase (Perkin Elmer, USA). The inner primers were used for the cycle-sequencing reaction, and fragments were separated by electrophoresis on an ABI PRISM genetic analyzer (Applied Biosystem).

### Statistical Analyses

All statistical analyses were performed using the SPSS software (SPSS-16.0 Inc., Chicago, IL, USA). Hardy-Weinberg equilibrium (HWE) for genotype distribution was performed using a *χ^2^* goodness-of-fit test. Analysis of covariance was used to examine the correlation between the baseline values and changes of BMI and *MTHFR* C677T site. Results were considered significant at two-tailed p < 0.05. P-values at two-tailed < 0.05 were considered significant.

## Results

### Participant Characteristics

A total of 387 patients were enrolled in the group, and 31 patients withdrew from this study due to poor efficacy (4 individuals), withdrawal of informed consent (2 individuals), violation of protocol (18 individuals), loss of follow-up (5 individuals), or other reasons (2 individuals). There were 356 patients who actually completed 8 weeks of medication, including 190 males and 166 females, with an average age of 30.5 ± 10.3 years. The mean therapeutic dose of risperidone was 4.8 ± 1.2 mg/d.

### HWE

According to the HWE, there was no significant difference between the observed value and expected value of genotype frequency for *MTHFR* C677T (rs1801133) polymorphism. Thus, it suggested that the subjects were collected randomly from the general population. The population was randomly mated, and there were neither migration nor risk of selection bias.

### Association Analysis of *MTHFR* C677T Polymorphism With Risperidone-Induced Weight Gain

**Figure 1:**
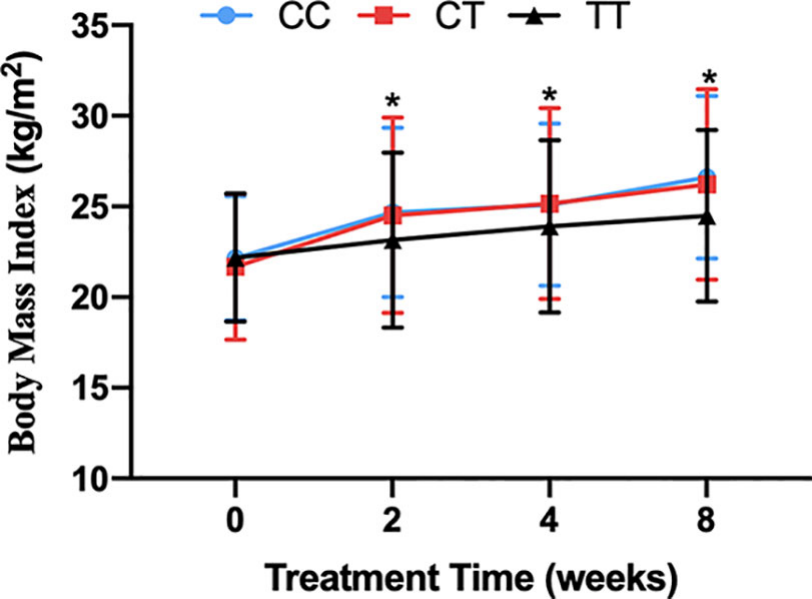
The BMI of carriers with CC, CT and TT genotypes of *MTHFR* C677T polymorphism during the 8-week risperidone treatment. There was no significant difference in BMI of three genotype carriers before risperidone treatment. However, the BMI of carriers with three genotypes of *MTHFR* gene increased over 2–8 weeks. After 2, 4, and 8 weeks of risperidone treatment, BMI added value of CC or CT genotype carriers was significantly higher than that of TT genotype carriers. Asterisks indicate that there are significant differences among BMI of CC, CT, and TT genotype carriers.

There was no significant difference in baseline BMI (kg/m^2^) before risperidone treatment among the three genotype carriers of *MTHFR* C677T polymorphism [CC (22.15 ± 3.43), CT (21.15 ± 4.02), TT (22.18 ± 3.53), F = 1.778, P = 0.169], but there were significant differences after 2, 4, and 8 weeks of treatment. The BMI of carriers with different genotypes of *MTHFR* gene increased over 2–8 weeks. After 8 weeks of risperidone treatment, BMI added value (kg/m^2^) of CC or CT genotype carriers was higher than that of TT genotype carriers, with statistically significant differences [8 weeks after treatment, BMI added value of CC (4.47 ± 1.09), CT (4.54 ± 1.27), TT (2.31 ± 0.75), F = 5.634, P = 0.001] (**Table 1** and **Figure 1**). Based on whether the rate of weight gain from baseline at 8 weeks of treatment exceeded 7%, it was divided into a weight gain group (n = 61) and a non-weight gain group (n = 295). The C allele frequency was significantly different between the two groups (48.4% vs 32.4%, *χ^2^* = 11.342, P = 0.001, OR = 1.93, 95% CI = 1.54–2.37) (**Table 2**).

**Table 1:** Association of *MTHFR* C677T polymorphism with risperidone-induced weight gain in schizophrenia.

	CC	CT	TT	*F (df = 2)*	*p-value*
N (%)	62 (17.4)	165 (46.5)	129 (36.1)	N.A.	N.A.
Sex (male/female)	32/30	84/81	65/64	0.987	0.464
Age (years)	24.2 ± 7.3	23.3 ± 6.8	22.7 ± 6.4	0.976	0.578
Baseline BMI (kg/m^2^)	22.15 ± 3.43	21.68 ± 4.02	22.18 ± 3.53	1.778	0.169
2-Week BMI Change from Baseline (kg/m^2^)	2.53 ± 0.32	2.84 ± 0.84	0.97 ± 0.14	5.412	<0.001
4-Week BMI Change from Baseline (kg/m^2^)	2.95 ± 1.12	3.48 ± 1.25	1.72 ± 0.66	6.398	<0.001
8-Week BMI Change from Baseline (kg/m^2^)	4.47 ± 1.09	4.54 ± 1.27	2.31 ± 0.75	5.634	0.001

df, degrees of freedom; N.A., not applicable.

**Table 2:** Comparison of *MTHFR* C677T genotype and allele frequency between groups with and without significant weight gain (%).

MTHFR C677T	Weight gain group n = 61	Non-weight gain group n = 295	χ^2^ (df)	p-value
**Genotype, n (%)**				
CC	15(24.6)	30(10.2)	12.099 (2)	0.002
CT	29(47.5)	131(44.4)		
TT	17(27.9)	134(45.4)		
**Allele, n (%)**				
C	59(48.4)	191(32.4)	11.342 (1)	0.001
T	63(51.5)	399(67.6)		

df, degrees of freedom.

## Discussion

Currently, atypical antipsychotic medications such as risperidone, olanzapine, and quetiapine are the first-line drugs for the treatment of schizophrenia. With their widespread use in clinical practice, the problem of drug-induced weight gain has received more and more attention (4). Until now, the specific mechanism of weight gain caused by antipsychotics is unclear. However, previous studies have shown that genetic factors may be closely related to this process. Different individuals may have large differences in weight gain even when taking the same drug of the same dose (5). There are a large number of reports about the susceptible genes related to weight gain caused by antipsychotics. However, these researches were mainly focused on candidate genes related to the target of antipsychotics, such as *DRD2* and *HTR2C* (8). Recently, there have been more and more studies about genes which affect energy metabolism and carbohydrate metabolism such as leptin and its receptor (*LEP*, *LEPR*) gene, and explore their association with weight gain caused by antipsychotics (24). These findings provide new ideas to study the mechanism of weight gain caused by antipsychotics.

This study suggested that, during the acute risperidone treatment, the *MTHFR* C677T C allele carriers were more likely to show a tendency to drug-induced weight gain than TT homozygous carriers. A previous study in 2014 analyzed the association of *MTHFR* C677T polymorphism with antipsychotic-induced weight gain for Chinese Han (n=182) and Spanish Caucasians (n=72) schizophrenia patients (15). It found that patients with the 677 CC genotype had a significantly greater BMI increasement compared to T-allele carriers in both Chinese and Spanish population. Even though this study found *MTHFR* polymorphism was associated with weight gain induced by antipsychotics, the sample size of this study was relatively small. Moreover, initial antipsychotic drug treatment for Chinese Han patients primarily consisted of risperidone (n=114) and chlorpromazine (n=60); only eight patients received clozapine, fluphenazine, or sulpiride. That meant the 182 schizophrenia patients received different types of antipsychotics. Therefore, the research about the association between *MTHFR* polymorphism and weight gain caused by single antipsychotics is rare. Our study confirmed that the individual differences in weight gain caused by risperidone, a first-line atypical antipsychotic, might be affected by *MTHFR* polymorphism.

The precise mechanism by which C677T polymorphism in the *MTHFR* gene to influence weight gain induced by risperidone remains unclear. As mentioned before, *MTHFR* exerts an important role in folate/homocysteine metabolism. The C677T polymorphism may decrease the activity of *MTHFR*, leading to the increase of serum homocysteine level. Homocysteine plays a key role in cell metabolism because it participates in the transfer of methyl groups in the activated methyl cycle. This cycle is in charge of global and gene promoter-specific DNA methylation, which is one of several epigenetic mechanisms involved in gene expression regulation (25, 26). Some researchers postulated that increased levels of homocysteine might influence weight gain *via* epigenetic control of gene expression in body fat storage regulation (27–29). Therefore, the possible mechanism is that *MTHFR* C677T polymorphism results in decreased DNA methylation of genes involved in the regulation of body weight (15).

However, this study has several limitations. Firstly, the sample size may not be sufficient. Therefore, further studies are needed to enroll more participants to confirm the present findings in our study. Secondly, side effects of drugs are not determined by a single gene, but by the interaction of multiple genes at different loci. Therefore, further studies are encouraged to explore the effect of gene-gene interaction on risperidone-induced weight gain. Thirdly, despite the influence of the genotype of SNP rs1801133 on weight gain induced by risperidone in schizophrenia, the specific mechanism is unclear. Moreover, our current results were limited to the Chinese Han population and we also did not further clarify the relationship between other SNPs in the *MTHFR* gene and risperidone-induced weight gain.

In conclusion, the *MTHFR* C677T polymorphism showed significant associations with risperidone-induced weight gain in 356 patients of Chinese Han ancestry treated with risperidone. Due to the large ethnic differences in *MTHFR* C677T polymorphism, the results of drug genetic association in the Chinese Han population might help clinical precision medicine in the future. If researches could find the relationship between antipsychotics and other susceptible genes, and further explore the relationship between drug-induced metabolic syndrome and cognitive and social function, it will be helpful for clinical application. In addition, the specific neurobiological mechanism of genetic polymorphisms affecting drug-induced weight gain needs to be further explored, and long-term follow-up, and validation in different and larger populations will be required in further studies.

## Data Availability Statement

The original contributions presented in the study are publicly available. This data can be found here: https://opendata.pku.edu.cn/dataset.xhtml?persistentId=doi:10.18170/DVN/FBWPVH.

## Ethics Statement

The studies involving human participants were reviewed and approved by the Ethical Committee of Institute of Mental Health, Peking University. The patients/participants provided their written informed consent to participate in this study.

## Author Contributions

WY provided the funds and designed the study. JL and NW analyzed the data and wrote the draft of the manuscript. MM recruited schizophrenia patients. TL collected peripheral blood samples. HY supervised this study.

## Funding

The study was funded by the National Key R&D Program of China (2016YFC1307000), National Natural Science Foundation of China (81825009, 81901358, 81221002), Peking University Clinical Scientist Program supported by “the Fundamental Research Funds for the Central Universities” (BMU2019LCKXJ012), Academy of Medical Sciences Research Unit (2019-I2M-5-006); PKUHSC-KCL Joint Medical Research (BMU2020KCL001), Beijing Science and Technology Commission (D171100007017002).

## Conflict of Interest

The authors declare that the research was conducted in the absence of any commercial or financial relationships that could be construed as a potential conflict of interest.
